# Assessment of Resection Margins in Bone Tumor Surgery

**DOI:** 10.1155/2020/5289547

**Published:** 2020-12-10

**Authors:** Corentin Malherbe, Bernard Crutzen, Jean Schrooyen, Giovanni Caruso, Frédéric Lecouvet, Christine Detrembleur, Thomas Schubert, Pierre-Louis Docquier

**Affiliations:** ^1^Service de Chirurgie Orthopédique et Traumatologique, Cliniques Universitaires Saint-Luc, Avenue Hippocrate 10, 1200 Brussels, Belgium; ^2^Département D'Imagerie Médicale, Cliniques Universitaires Saint-Luc, Avenue Hippocrate 10, 1200 Brussels, Belgium; ^3^Neuro Musculo Skeletal Lab (NMSK), Institut de Recherche Expérimentale et Clinique, Université Catholique de Louvain, Secteur des Sciences de la Santé, Avenue Mounier 53, B-1200 Brussels, Belgium

## Abstract

Limb salvage surgery is now the preferred procedure for bone tumor surgery. To decrease the risk of local recurrence, it is crucial to obtain adequate resection margins. The obtained margins must be evaluated postoperatively because they influence what treatment is given subsequently when margins are not adequate (e.g., surgical revision and radiotherapy). The study aims to evaluate margin assessment of tumor specimen by MRI compared to conventional histology (to establish the viability of using MRI) and assess the accuracy of a patient-specific instrument when narrow margins were aimed. The resection margins in 12 consecutive patients that were operated on for bone tumor resection were prospectively analyzed using three methods: MRI of the resection specimen, macroscopic evaluation of specimen slices, and microscopic pathological evaluation. The assessments were qualitative (R0, R1, and R2) and quantitative (distance in mm). MRI, macroscopic, and microscopic margins generated similar results for both the qualitative (all resections were R0) and quantitative assessments. The median error in safe margins was 2 mm with a surgical guide (PSI) and 5 mm without a surgical guide. Local recurrences were not detected after a mean follow-up period of 3.7 years (range, 2.1–5 years); however, four patients died during the study. In conclusion, MRI is a valuable tool for assessing safe margins. When specimens are not available for pathological assessment (e.g., extracorporeally irradiated autograft or autoclaved autograft), MRI could be used to evaluate margins. In particular, when tumor volume is high, MRI could also help to focus the pathological examination on areas of concern.

## 1. Introduction

When possible, limb salvage is the preferred procedure to manage malignant bone tumors. In such cases, “en bloc” surgical resection is required without penetrating the tumor [1–6]. During the procedure, the surgeon resects the tumor while retaining a safe continuous margin of healthy tissues around it. Precise preoperative planning is required to respect margins (without penetrating the tumor) and to avoid cutting neurovascular structures. No universal quantitative recommendation is available on optimal margin size in the published literature. [7] The qualitative recommendation for margins is R0 resection, [8] which is when no residual tumor remains in the patient. R1 (removal of all macroscopic disease, but microscopic margins are positive for tumor) and R2 (gross residual tumor that was not resected) resections are clearly linked to a worse prognosis; [9] consequently, a second surgery or adjuvant radiotherapy is necessary [10].

Currently, the pathological examination of bone tumor takes time for voluminous tumors. The time necessary to decalcify, embed in paraffin, and analyze the specimen is ten to fifty days. Furthermore, obtaining an accurate analysis of the whole specimen is often difficult. The postoperative evaluation of resection margins by MRI [11–13] has been proposed in the published literature. MRI evaluation is more rapid and can be used to assess the whole specimen easily, including those with voluminous tumors. The study objectives are as follows:

1. To assess the value of an MRI and its accuracy in assessing the margins of the resection specimen
2. To assess the accuracy of a patient-specific instrument (PSI) in obtaining the planned margin

## 2. Materials and Methods

### 2.1. Ethics Committee Agreement

Agreement of the central ethics committee was obtained and was given the following number: UCL/MGS/001 (2009/02 AVR/126).

### 2.2. Patient Series

Data were obtained prospectively between January 2015 and January 2017. Based on our protocol, inclusion criteria were primary bone sarcoma accessible to resection surgery. There were five osteosarcomas, six Ewing's sarcomas, and one fibrous dysplasia. Diagnosis of fibrous dysplasia was only possible postoperatively, as imaging was unclear; thus, the decision to perform resection was made without a previous biopsy. Localizations were two iliac bones, six femurs, one ulna, two radiuses, and one fibula. Three patients were already metastatic at the time of tumor discovery. The time between surgery and the last follow-up was 3.7 years (range: 2.1–5 years). Four patients died in the two postoperative years (Table 1).

### 2.3. Preoperative MRI Evaluation

Two preoperative MRIs of the bone tumor were obtained in all cases (with general anesthesia for children): prechemotherapy MRI (within one month before the first course of chemotherapy) and postchemotherapy MRI (at the end of chemotherapy and before surgery). A Siemens MAGNETOM Verio 3 Tesla MRI was used, and the following specific sequences were used for the preoperative exams: axial 3D T1- and 3D T2-weighted images, coronal proton density-weighted images with fat saturation, and axial and sagittal T1-weighted images with gadolinium enhancement were obtained. MRI acquisition parameters were specified as follows: reconstruction matrix 176 × 176, 0.5 mm section thickness and 0.5 mm spacing between slices. The MRIs were reconstructed by multiplanar reconstruction (MPR) and were saved in DICOM format with a picture archiving and communication system (PACS; Carestream Health, NY, USA).

### 2.4. Surgery

Surgery was executed with PSI in seven cases and without PSI in five cases. In seven cases, a 1 cm margin was planned to preserve a joint or an epiphysis, and a PSI was used (to increase the accuracy of the resection margins [14, 15]). PSI was also used in one case where a step-cut was planned to increase stability and for allograft cutting in another patient.

When the planned margin exceeded 2 cm, PSI was not used. This was the case, for example, when a minimal 17 cm resection was needed to implant a growth prosthesis. The PSI was designed by an engineer (3D-Side®). The tumor was delineated on MRI using the software developed by 3D-Side®. The preferred MRI used to delineate the tumor type was T1 [16]. The CT scanner of the preoperative PET scanner was used with minimal 1 mm section thickness. Tumor volume obtained from MRI was merged with the CT scanner to create a 3D scan in which the tumor was visible (by using the software developed by 3D-Side). The PSI was created from the 3D scanner based on the margins selected by the surgeon. After tumor resection, the specimen was oriented using surgical threads (craniocaudal). A sample was collected from the tumor for genetic analysis, and the tumor was immersed in 4% formalin.

### 2.5. Postoperative MRI Evaluation of the Resection Specimen

**Figure 1:**
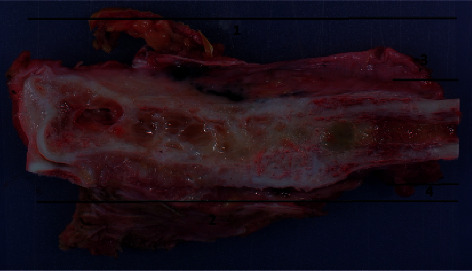
A 36-year-old patient with Ewing's sarcoma of the distal ulna. Example of schematic oriented representation of margins to be measured. The corresponding MRI slices (created by MPR) were given for measures.

**Figure 2:**
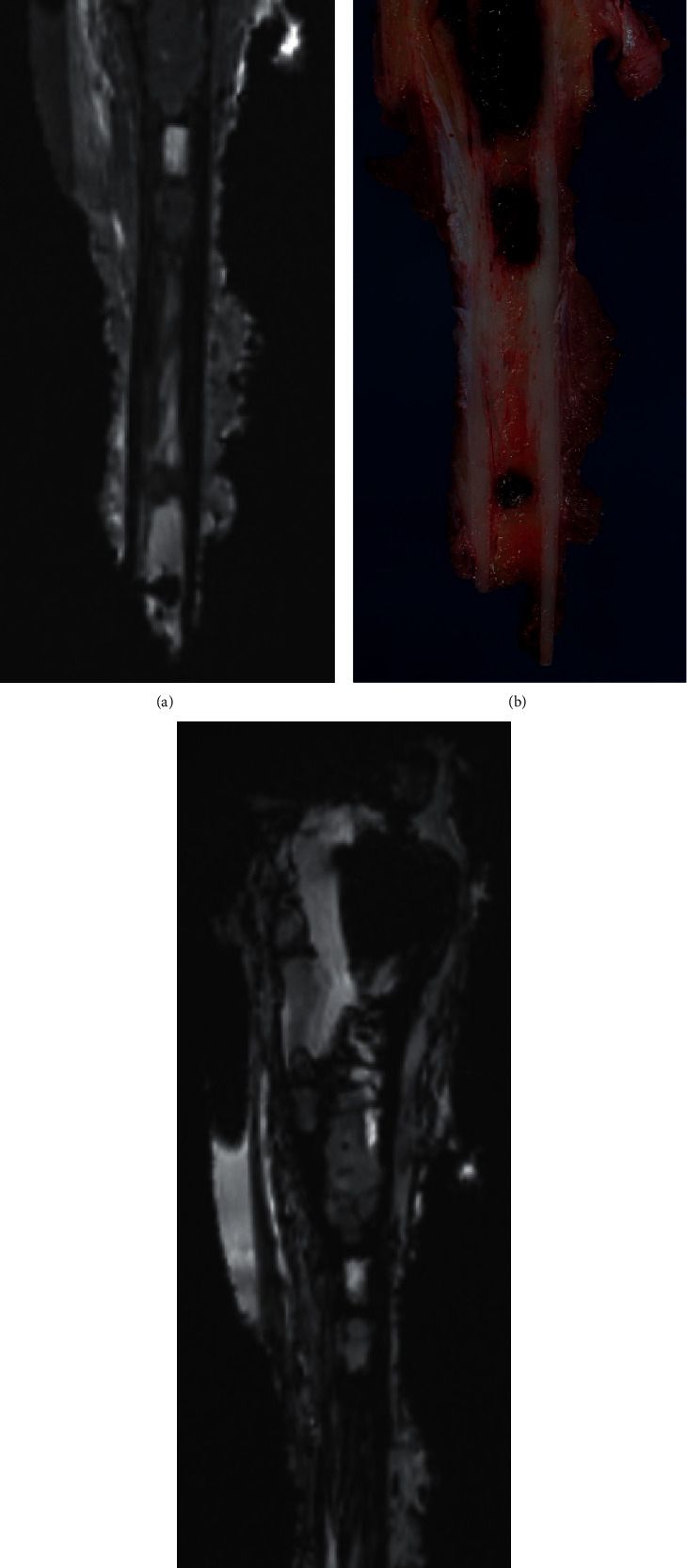
A 9-year-old girl with osteosarcoma of the distal radius. A step-cut was performed proximally for the stability of surgical reconstruction by using a PSI. Two margins were measured (medial and lateral part of the step-cut). Note the difficulty to visualize the cortical bone in the step-cut with MRI. (a) T1-weighted MRI; (b) macroscopic cut; (c) T2-weighted MRI.

**Figure 3:**
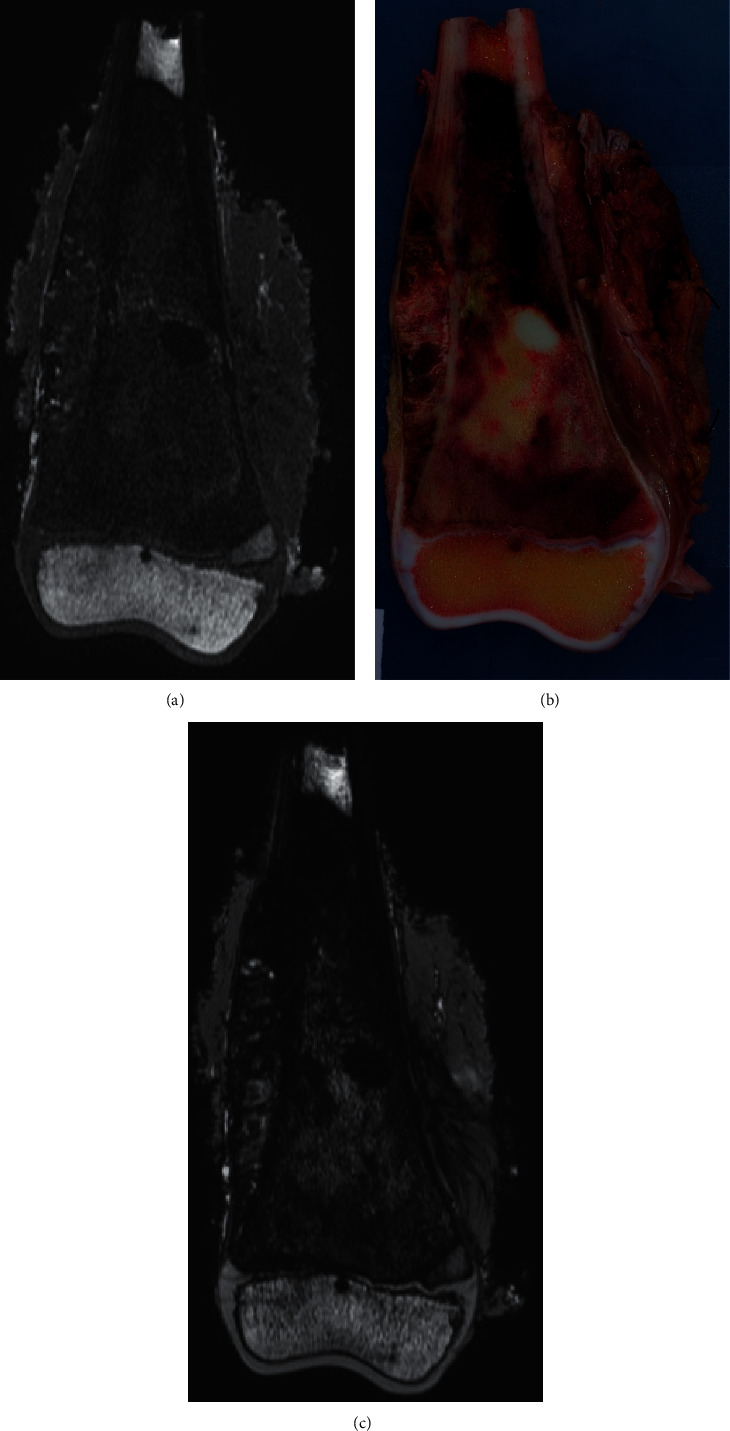
An 11-year-old boy with distal femur osteosarcoma. No PSI was used because 17.5 cm bone resection was necessary for a growth prosthesis. One margin was measured. (a) T1-weighted MRI; (b) macroscopic cut; (c) T2-weighted MRI.

MRIs of the resected tumor specimen were obtained using a Philips Achieva 3 Tesla MRI. The specific sequences were axial 3D T1- and 3D T2-weighted MRI. The MRI was performed within 6 h of surgery. The same MRI machine was used for the entire series. Specimens were oriented according to the long axis of the long bone or the craniocaudal axis of the pelvic bone. MRI acquisition parameters were specified as follows: reconstruction matrix 176 × 176, 0.5 mm section thickness, and 0.5 mm spacing between slices. The MRIs were reconstructed by MPR and were saved in DICOM format with PACS (Carestream Health, NY, USA). Through MPR, MRI slices were created similar to the macroscopic cuts. MRIs were analyzed, classified, and measured by three independent observers (i.e., blindly) based on the standardized classification of the Union for International Cancer Control (UICC) [17]. Observers had access to the preresection MRI to help analyze the specimen. The three observers included an experienced radiologist used to tumor imaging, a junior radiologist with no experience in bone tumor surgery, and an experienced orthopedic surgeon used to tumor surgery. UICC classification distinguished R0 with adequate safe margins (>1 mm), R1 as possible microscopic residuals (minimal margin between 0 and 1 mm), and R2 as macroscopic residual disease (<0 mm). Examiners received a folder with schematic oriented representations of the margins and axes to use for making measurements (Figure 1). Measurements were requested for each saved MRI cut (T1 and T2) (equivalent to the macroscopic cuts) (Figures 2 and 3) and for the whole MRI.

### 2.6. Macroscopic Evaluation

**Figure 4:**
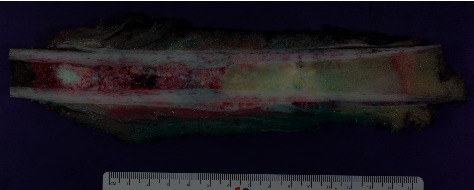
Macroscopy of femoral osteosarcoma with two cuts, one horizontal and one step-cut.

Within the hour following MRI (i.e., before cutting), the specimen was inked. The specimen was sliced in a coronal plane using a band saw. Each slice was oriented and numbered in ascending number from posterior to anterior. A picture of each section was captured with a graduated scale in the same layout (Figure 4). The size and margins of the specimen were measured by an orthopedic resident and were expressed in millimeters. The specimens were subsequently used for macroscopic analysis by both the pathologist and orthopedic resident based on UICC classification. After macroscopic analysis, the specimen was immersed in 4% formalin.

### 2.7. Pathological Evaluation

Determination of surgical margins by pathologists is now standardized [18, 19]. A second macroscopic evaluation of the tumor margin was performed by the pathologist to locate areas with the narrowest margins. Tissue samples were taken at these locations for histology. These blocs were embedded in paraffin and cut to a thickness of 5 microns before staining with Hematoxylin and Eosin. The distance between the inked border and tumor was measured microscopically. The measure was expressed in millimeters. The pathologist again evaluated the quality of the resection based on the UICC classification, using none of the postoperative MRI information. The pathologist rated the type, grade [20], and stage of resection.

### 2.8. Statistical Analysis

Macroscopic measurements (which are considered as the gold standard) were compared with the MRI measurements of the three independent observers. All statistical analyses were performed using SigmaPlot software 13.0. A Kruskal–Wallis test (one-way analysis of variance on ranks) was used to evaluate agreement among the observers. We also used the Bland and Altman method and Passing–Bablok regression to compare and calculate the bias of each value obtained by the three observers and macroscopy. Using Passing–Bablok regression, intercept A represented the measure of the systematic differences between values. If the confidence interval for intercept A did not contain that value 0, it was concluded that A was significantly different from 0 and that both methods differed, at least, by a constant amount. Slope B was the measure of the proportional difference between values. If the confidence interval for slope B did not contain value 1, it was concluded that B was significantly different from 1 and that there was at least a proportional difference between the two methods. We checked the correlation between measures using Spearman's correlation. To compare planned safe margins and the actual achieved margins with and without PSI, we evaluated the difference between the four independent measurements and the planned distance (in the operating protocol). We calculated the mean, median, maximum error, and standard deviation. For example, if it was planned to cut the distal femur at 175 mm from the medial condyle, we checked whether that distance was 175 mm. To compare planned safe margins with PSI, we compared the planned margins with the smallest obtained margins. For example, if a 10 mm margin was planned and the smallest margin was measured at 8 mm, the error was 2 mm.

## 3. Results

Agreement among MRI, macroscopic, and pathological evaluation was perfect for the qualitative assessment. All bone resections were evaluated as being R0 using all three methods. There was no statistically significant difference between the MRI evaluation by the three observers (senior radiologist, junior radiologist, and senior orthopedist) versus the macroscopic evaluation (based on 145 measures) (*p*=0.94) (Table 2).

A very strong correlation was found between the three observers. A very strong correlation was also found between macroscopy and junior radiologist and senior orthopedist (Table 3). A strong correlation was found between the senior radiologist and macroscopy.

If the confidence interval for intercept A did not contain the value 0, it was concluded that A was significantly different from 0 and both methods differed at least by a constant amount. If the confidence interval for slope B did not contain the value 1, it was concluded that B was significantly different from 1 and there was at least a proportional difference between the two methods.

The bias between methods was very small; however, Passing–Bablok regression indicated that the methods were not equal (Table 4).

When PSI was not used, the median and maximum margins of error (compared to the planned margin) were 5 mm and 11 mm, respectively (Table 5).

When using PSI, the median and maximum margins of error were 2 mm and 6 mm, respectively (Tables 6 and 7). This calculation was made on five patients. One patient had more than the expected margin because the volume of the tumor had reduced by a few centimeters during chemotherapy. For another patient (patient 5), the cut was performed accurately in accordance with the plan and PSI, but the measured margin was 9 mm less than expected due to a mistake in surgical planning. Fortunately, the margin was still R0. This result was caused by the bad resolution of the prechemotherapy MRI, inducing an error in the volume planning of the tumor.

## 4. Discussion

Quantitative comparison between MRI and macroscopic evaluations of resection specimens is not available in the published literature. All existing MRI studies compared tumor size before surgery and macroscopically [21–23]. These studies demonstrated a systematic error between the two methods ranging from 5.9 to 19 mm. The current study also obtained similar margins of error when comparing MRI to macroscopic assessment. The current prospective study followed the same protocol and the same conditions (the same MRI was used). The MRI and macroscopic measurements were carried out by the three readers, including a junior orthopedist, to reduce human and material bias.

The MRI evaluation was performed in thirty minutes, and the radiologist assessment took no more than fifty minutes. If rapid access to MRI is possible, the full analysis can be done in less than an hour. Therefore, this MRI assessment is more rapid than the classical pathological assessment (paraffin-embedded sections) (10 to 15 days). A frozen section is possible but cannot be used in situations like ECRT where the bone is reimplanted. It also can have a sampling error as the entire margin cannot be assessed.

For MRI, the presence of a sterile plastic wrap did not alter the picture quality of MRI because no artifact was generated by the plastic wrap.

The space between slices was 0.5 mm when measured using the MRI and 3–4 mm when measured macroscopically under the best conditions (slices by band saw). MRI facilitated the use of thinner slices and provided much more information, such as skip metastasis and small lesions. MRI specimens can be compared to preoperative MRIs to determine the location of the lesion more accurately. However, the pathological analysis of the resected specimen provides information that MRI cannot provide, such as the percentage of tumor necrosis. Thus, pathological analysis is required when this information is needed to adjust adjuvant chemotherapy protocols. When tumor specimens are not available for pathology assessment, MRI is the optimal tool for assessing surgical margins, such as the extracorporeal irradiation of specimens, reimplantation, and autoclaved autografts. MRI also complements in-depth pathological analyses of entire tumor specimens, highlighting questionable areas for pathologists.

Some issues were encountered when analyzing the margins with MRI. When the cortical bone is not surrounded by soft tissue, it cannot be distinguished by MRI. Soft tissue tends to retract after the bone is cut and when immersed in formalin (Figure 2). Often, the cortical bone at the site of the section is not surrounded by soft tissue but by air; consequently, it cannot be distinguished easily (black on black in an MRI). The MRI tended to minimalize the margin in comparison to macroscopy. The safe margin tends to be 1.5 mm less than the planned range due to the thickness of the saw blade. Some bone material is also lost when a bone is cut due to the vibration of the saw blade (called the kerf effect) [24].

The senior radiologist recorded smaller margins compared to the junior radiologist and senior orthopedist (Table 2). This difference could be explained by the method used by the senior radiologist. The senior radiologist interpreted the MRI in its entirety by visualizing the slices before and after they were selected. If the tumor was closer to the margin on an adjacent slice, the senior radiologist measured the margin accordingly. This approach might explain the systematically smaller margin recorded by the senior radiologist. In comparison, the orthopedist and junior radiologist selected slices without evaluating adjacent slices.

When using PSI, the median error was 2 mm (better accuracy compared to without PSI); however, sufficient planning is required. When planning is inadequate (i.e., the tumor is not appropriately delineated on MRI), there is a systematic effect on the final measurement of the margin. Thus, meticulous analysis of the preoperative MRI is required to delineate the limit of the tumor. If the quality of the preoperative exam is poor, it should be repeated.

The accuracy of MRI in assessing the bone extension of tumors was assessed by Thompson et al. [16]. The authors obtained a difference of 13 mm for the mean tumor size when comparing MRI to histopathology. We did not obtain this difference in our four measurements. The human factor in surgery is always present. Thus, for various reasons, using a margin that is too narrow is dangerous. In general, the bone margin is planned based on the prechemotherapy MRI, whereas the soft tissue margin is planned based on the postchemotherapy MRI. Some recent studies also suggested planning bone margins based on postchemotherapy MRI [25]. We can overestimate the bone margin if the tumor shrinks during chemotherapy. To exclude the chance that mistakes in cutting were masked by a decline in tumor size, we also checked the size of the specimens (planned and achieved) when comparing the two results. Tumor volume only declined in one case; thus, this case was excluded from the analysis measuring PSI accuracy. In the series without PSI, we only checked the accuracy of specimen size.

This study has some limitations. We did not compare T1 and T2 measurements. Some studies showed closer margins with T1-weighted measurements. To assess the marginal cortex with greater precision, T1 and T2 are necessary. All of the histologic analyses were performed by a single observer and only once. Only 12 patients were included in the study, of which seven benefited from PSI.

## 5. Conclusions

This study shows that the MRI evaluation of the resection specimen was equivalent to the pathological evaluation, both qualitatively and quantitatively. MRIs are limited in that they cannot inform the pathologist on the extent of necrosis and type of tumor. When this information is needed, MRI cannot replace pathological evaluation. Surgical resection with PSI was more accurate than that without PSI (median error of 2 mm versus 5 mm); however, the series was too small to generate recommendations on margins. Despite PSI, meticulous preoperative planning on MRIs remains mandatory. To preserve a joint or an epiphysis, PSI is recommended to decrease the margin.

## Figures and Tables

**Table 1:** Patients' data.

*N*	Age (years) ^*∗*^	Gender	Tumor location	Type	Surgical guide (PSI)	Presence of metastases	Evolution
1	11	M	Femur	OS	Yes	No	NED
2	11	F	Pelvis	EwS	Yes	No	NED
3	21	F	Femur	FD	Yes	No	NED
4	9	F	Radius	OS	Yes	No	NED
5	7	F	Radius	EwS	Yes	No	NED
6	6	F	Femur	EwS	No	No	NED
7	12	M	Femur	OS	No	Yes	Deceased
8	9	M	Femur	OS	No	No	NED
9	11	M	Femur	OS	No	No	NED
10	36	M	Ulna	EwS	No	Yes	Deceased
11	14	M	Pelvis	EwS	Yes	No	Deceased
12	17	M	Fibula	EwS	Yes	Yes	Deceased

*M*: male; *F*: female; NED: no evidence of disease; OS: osteosarcoma; EwS: Ewing's sarcoma; FD: fibrous dysplasia.  ^*∗*^At the time of surgery.

**Table 2:** Equivalence between MRI and macroscopic evaluation (Kruskal–Wallis one-way analysis of variance on ranks) (*n* = 145).

	Median (range) in mm
MRI senior radiologist	20 [14–26.5]
MRI senior orthopedist	21 [15–29]
MRI junior radiologist	20.5 [13.8–26]
Macroscopy	22 [13–26]

**Table 3:** Differences between the methods according to Passing–Bablok regression.

	Intercept A [CI]	Slope B [CI]	Correlation coefficient
MRI senior radiologist/senior orthopedist	−1.8 [−3 to 0]	1.1 [1 to 1.1]	0.888 (*p* < 0.0001)
MRI senior radiologist/macroscopy	−2.1 [−4.8 to -0.3]	1.1 [1.0 to 1.2]	0.736 (*p* < 0.0001)
MRI senior orthopedist/macroscopy	−2.9 [−4.5 to -1.4]	1.1 [1.1 to 1.2]	0.896 (*p* < 0.0001)
MRI senior radiologist/junior radiologist	0 [−1.1 to 0]	1 [1.0 to 1.0]	0.882 (*p* < 0.0001)
MRI senior orthopedist/junior radiologist	1.3 [0.4 to 2.1]	0.9 [0.9 to 0.97]	0.935 (*p* < 0.0001)
MRI junior radiologist/macroscopy	−0.8 [−1.8 to 0]	1.0 [1.0 to 1.1]	0.901 (*p* < 0.0001)

**Table 4:** Bias between the methods.

	Bias (mm)	Standard deviation	Margin bigger for
MRI senior radiologist/senior orthopedist	1.19	7.3	Orthopedist
Macroscopy/MRI senior radiologist	−1.37	8.5	Macroscopy
MRI senior orthopedist/macroscopy	0.11	5.7	Macroscopy
MRI senior radiologist/junior radiologist	1.26	6.9	Junior radiologist
MRI senior orthopaedist/junior radiologist	0.16	4.15	Junior radiologist
MRI junior radiologist/macroscopy	−0.49	4.86	Junior radiologist

**Table 5:** Reliability without PSI (in mm).

	Median	Max	Std dev
MRI senior radiologist	5	8	2.6
MRI senior orthopedist	6	11	3.7
MRI junior radiologist	3	7	2.4
Macroscopy	8	10	3.5
ALL	5	11	3.0

**Table 6:** Reliability with PSI (in mm) with patient 5.

	Median	Max	Std dev
MRI senior radiologist	−2	−9	3.6
MRI senior orthopedist	−2	−6	2.2
MRI junior radiologist	−2	−9	2.7
Macroscopy	−2	−9	3.9
ALL	−2	−9	3.1

**Table 7:** Reliability with PSI (in mm) without patient 5.

	Median	Max	Std dev
MRI senior radiologist	−2	−3	0.8
MRI senior orthopedist	−2	−6	2.0
MRI junior radiologist	−2	−3	0.7
Macroscopy	−2	−3.5	1.3
ALL	−2	−6	1.2

## Data Availability

Due to the specific nature of the pathology and the limited number of cases, it is easy to identify patients despite the anonymization of the data. These data will be shared on request from members of the medical profession. The statistical data used to support the findings of this study are available from the corresponding author upon request. The measurement data used to support the findings of this study are available from the corresponding author upon request.

## Supplementary Materials

Supplementary Materials The statistical data and the measurement data are available on a statistic.docx and measurementdata.xls. These files are also available from the corresponding author upon request.
