# Supplementary material for: Assessment of Resection Margins in Bone Tumor Surgery
Source: Sarcoma. 2020 Dec 10;2020:5289547. doi: 10.1155/2020/5289547 (PMC7789471; doi:10.1155/2020/5289547)
Supplement: Supplementary Materials — The statistical data and the measurement data are available on a statistic.docx and measurementdata.xls. These files are also available from the corresponding author upon request. [file 5289547.f1.zip › 5289547/statistic.docx]

Dr. Corentin Malherbe

1. **Compared margin for 3 measure ; via Kruskal-Wallis One Way Analysis of Variance on Ranks**

**A : MRI senior radiologist**

**B : MRI senior orthopedist**

**C : Macroscopy**

**D : MRI junio radiologist**

**Kruskal-Wallis One Way Analysis of Variance on Ranks (n=145)**

|  | Median [range]  in mm |
| --- | --- |
| A | 20 [14-26.5] |
| B | 21 [15-29] |
| C  D | 22 [13-26]  20.5 [13.8-26] |

P=0.94

No difference between the 4 technique to evalued margins. On the median and with overall calcul.

1. **Bland and Altman et Passing Bablok regression**

- **Systematic differences**. The intercept A is a measure of the systematic differences between the two methods. The 95% confidence interval for the intercept A can be used to test the hypothesis that A=0. This hypothesis is accepted if the confidence interval for A contains the value 0. If the hypothesis is rejected, then it is concluded that A is significantly different from 0 and both methods differ at least by a constant amount.
- **Proportional differences**. The slope B is a measure of the proportional differences between the two methods. The 95% confidence interval for the slope B can be used to test the hypothesis that B=1. This hypothesis is accepted if the confidence interval for B contains the value 1. If the hypothesis is rejected, then it is concluded that B is significantly different from 1 and there is at least a proportional difference between the two methods.

**2.1 Difference between senior radiologist et orthopedist senior**

**A : MRI senior radiologist – method 1**

**B : MRI senior orthopedist – method 2**

Biais 1.18 (7.3) mm : margin little bigger for orthopedist

| **Systematic differences** | |
| --- | --- |
| Intercept A | -1.8333 |
| 95% CI | -3.0000 to 0.0000 |
| **Proportional differences** | |
| Slope B | 1.0833 |
| 95% CI | 1.0000 to 1.1429 |
| **Random differences** | |
| Residual Standard Deviation (RSD) | 5.1854 |
| ± 1.96 RSD Interval | -10.1634 to 10.1634 |
| **Linear model validity** | |
| Cusum test for linearity | No significant deviation from linearity (P=0.71) |

**Spearman rank correlation coefficient**

| Correlation coefficient | 0.888 |
| --- | --- |
| Significance level | P<0.0001 |
| 95% CI | 0.847 to 0.919 |

Good correlation

Reject H0. Conclude that the methods are not equal.

**2.2 Difference between MRI senior radiologist A and C Par macroscopy**

**A : MRI senior radiologist**

**C : macroscopy**

Biais -1.3 (8.5) mm : margin little smaller for radiologist

| **Systematic differences** | |
| --- | --- |
| Intercept A | -2.1250 |
| 95% CI | -4.7778 to -0.3125 |
| **Proportional differences** | |
| Slope B | 1.1250 |
| 95% CI | 1.0208 to 1.2222 |

**Spearman rank correlation coefficient**

| Correlation coefficient | 0.736 |
| --- | --- |
| Significance level | P<0.0001 |

Moderate correlation

Reject H0. Conclude that the methods are not equal.

**2.3 Difference B MRI senior orthopedist et C macroscopy**

**B : MRI senior orthopedist**

**C : macroscopy**

Biais 0.11 (5.7) mm : small difference macro vs ortho MRI

| **Systematic differences** | |
| --- | --- |
| Intercept A | -2.9487 |
| 95% CI | -4.5455 to -1.4000 |
| **Proportional differences** | |
| Slope B | 1.1282 |
| 95% CI | 1.0667 to 1.1818 |
| **Random differences** | |
| Residual Standard Deviation (RSD) | 3.7255 |
| ± 1.96 RSD Interval | -7.3020 to 7.3020 |
| **Linear model validity** | |
| Cusum test for linearity | No significant deviation from linearity (P=0.53) |

**Spearman rank correlation coefficient**

| Correlation coefficient | 0.896 |
| --- | --- |
| Significance level | P<0.0001 |
| 95% CI | 0.856 to 0.926 |

Good correlation

Reject H0. Conclude that the methods are not equal.

**2.4 Difference between MRI senior Rx et MRI junior RX**

Biais 1.26 (6.9) : margin bigger for MRI RX junior

| **Systematic differences** | |
| --- | --- |
| Intercept A | 0.0000 |
| 95% CI | -1.0682 to 0.0000 |
| **Proportional differences** | |
| Slope B | 1.0000 |
| 95% CI | 1.0000 to 1.0455 |
| **Random differences** | |
| Residual Standard Deviation (RSD) | 4.9806 |
| ± 1.96 RSD Interval | -9.7619 to 9.7619 |
| **Linear model validity** | |
| Cusum test for linearity | No significant deviation from linearity (P=0.51) |

**Spearman rank correlation coefficient**

| Correlation coefficient | 0.882 |
| --- | --- |
| Significance level | P<0.0001 |
| 95% CI | 0.838 to 0.914 |

Good correlation

Reject H0. Conclude that the methods are not equal.

**2.5 Difference between MRI senior ortho vs MRI junior RX**

Biais 0.16 (4.15) few differences entre ORTHO et RX junior

| **Systematic differences** | |
| --- | --- |
| Intercept A | 1.3143 |
| 95% CI | 0.3958 to 2.1818 |
| **Proportional differences** | |
| Slope B | 0.9429 |
| 95% CI | 0.9091 to 0.9792 |
| **Random differences** | |
| Residual Standard Deviation (RSD) | 2.7479 |
| ± 1.96 RSD Interval | -5.3859 to 5.3859 |
| **Linear model validity** | |
| Cusum test for linearity | No significant deviation from linearity (P=0.86) |

**Spearman rank correlation coefficient**

| Correlation coefficient | 0.935 |
| --- | --- |
| Significance level | P<0.0001 |
| 95% CI | 0.910 to 0.953 |

Good correlation

Reject H0. Conclude that the methods are not equal.

**2.6 Macro vs MRI junior RX**

Biais 0.49 (4.86) : margin slighty larger for RX junior

| **Systematic differences** | |
| --- | --- |
| Intercept A | -0.8093 |
| 95% CI | -1.8000 to 0.0000 |
| **Proportional differences** | |
| Slope B | 1.0458 |
| 95% CI | 1.0000 to 1.0857 |
| **Random differences** | |
| Residual Standard Deviation (RSD) | 3.4884 |
| ± 1.96 RSD Interval | -6.8372 to 6.8372 |
| **Linear model validity** | |
| Cusum test for linearity | No significant deviation from linearity (P=0.69) |

**Spearman rank correlation coefficient**

| Correlation coefficient | 0.901 |
| --- | --- |
| Significance level | P<0.0001 |
| 95% CI | 0.863 to 0.929 |

Good Correlation

Reject H0. Conclude that the methods are not equal

Very good correlation betwen measurement and good correlation RX senior et MACRO

No CONVERGENCE BETWEEN THE TECHNIQUES despite low biases. Systematic error and proportionnal difference. (Human biais).

1. **Comparison in the assessment of margins for 4 measurement with and without PSI : Anova 2 factors**

|  | with PSI(mm) | Without PSI (mm) |
| --- | --- | --- |
| MRI senior radiologist | 172.5 (37.4) | 134.4 (40.1) |
| MRI junior radiologist | 174.5 (40.2) | 136.2 (40.9) |
| MRI orthopedist | 171.8 (41.4) | 133.6 (39.7) |
| Macro | 179.5 (40.9) | 135.6 (38.9) |
| Planned | 173.6 (38.9) | 137.0 (42.8) |

Pvaleur between 4 mesures : 0.99

Pvaleur measures x PSI : 0.99

1. Accuracy

| With PATIENT #5 |  |  |  |  |  |  |  |
| --- | --- | --- | --- | --- | --- | --- | --- |
|  | Mean | SD | Max | Min | Median | 25% | 75% |
| MRI Senior ortho | -3.0 | 2.2 | -1 | -6 | -2 | -6 | -1 |
| MRI Senior rx | -3.7 | 3.6 | -1 | -9 | -2 | -6.5 | -1.5 |
| MRI Junior rx | -2.8 | 2.7 | -1 | -9 | -2 | -3.3 | -1 |
| MACRO | -4.4 | 3.9 | -1 | -9 | -2 | -10 | -2 |
| ALL | -3.4 | 3.1 | -1 | -9 | -2 | -3.8 | -1.5 |
|  |  |  |  |  |  |  |  |
| Without PATIENT #5 |  |  |  |  |  |  |  |
|  | Mean | SD | Max | Min | Median | 25% | 75% |
| MRI Senior ortho | -2.6 | 2.0 | -1 | -6 | -2 | -4.5 | -1 |
| MRI Senior rx | -1.8 | 0.8 | -1 | -3 | -2 | -2.5 | -1 |
| MRI Junior rx | -2.7 | 2.8 | -1 | -3 | -2 | -2.5 | -1 |
| MACRO | -2.2 | 1.3 | -1 | -3.5 | -2 | -3.5 | -1 |
| ALL | -2.4 | 2.1 | -1 | -3 | -2 | -3 | -1 |
|  |  |  |  |  |  |  |  |
|  |  |  |  |  |  |  |  |
| précision avec guide | Mean | Std Dev | Max | Min | Median | 25% | 75% |
| MRI Senior ortho | 3.6 | 4.6 | 15.0 | 0 | 2.5 | 0 | 5.3 |
| MRI Senior rx | 2.7 | 3.7 | 12.0 | 0 | 2.0 | 0 | 3.5 |
| MRI Junior rx | 2.1 | 1.9 | 6.0 | 0 | 2 | 0 | 3.3 |
| MACRO | 4.8 | 4.8 | 11.0 | 0 | 3.5 | 0 | 10.25 |
| ALL | 3.3 | 3.9 | 15.0 | 0 | 2.0 | 0 | 5.0 |
|  |  |  |  |  |  |  |  |
| précision sans guide | Mean | Std Dev | Max | Min | Median | 25% | 75% |
| MRI Senior ortho | 6.2 | 3.7 | 11.0 | 1.0 | 6 | 3 | 9.5 |
| MRI Senior rx | 5.4 | 2.6 | 8.0 | 2.0 | 5 | 3 | 8 |
| MRI Junior rx | 3.6 | 2.4 | 7.0 | 1 | 3 | 1.5 | 6 |
| MACRO | 6.2 | 3.5 | 10.0 | 2 | 8 | 2.5 | 9 |
| ALL | 5.4 | 3.0 | 11.0 | 1 | 5 | 2.25 | 8 |
